# Real-Time Detection of Meningiomas by Image Segmentation: A Very Deep Transfer Learning Convolutional Neural Network Approach

**DOI:** 10.3390/tomography11050050

**Published:** 2025-04-24

**Authors:** Debasmita Das, Chayna Sarkar, Biswadeep Das

**Affiliations:** 1 Department of Computer Science and Engineering, Vellore Institute of Technology, Vellore Campus, Tiruvalam Road, Katpadi, Vellore 632014, Tamil Nadu, India; debasmita.das.1308@gmail.com; 2 Department of Clinical Pharmacology and Therapeutics, North Eastern Indira Gandhi Regional Institute of Health and Medical Sciences (NEIGRIHMS), Mawdiangdiang, Shillong 793018, Meghalaya, India; chayna_sarkar@hotmail.com; 3 Department of Pharmacology, All India Institute of Medical Sciences (AIIMS), Virbhadra Road, Rishikesh 249203, Uttarakhand, India

**Keywords:** brain tumor, meningioma, convolutional neural networks (CNNs), very deep transfer learning, VGG-16, magnetic resonance imaging, machine learning, radiomics

## Abstract

Background/Objectives: Developing a treatment strategy that effectively prolongs the lives of people with brain tumors requires an accurate diagnosis of the condition. Therefore, improving the preoperative classification of meningiomas is a priority. Machine learning (ML) has made great strides thanks to the development of convolutional neural networks (CNNs) and computer-aided tumor detection systems. The deep convolutional layers automatically extract important and dependable information from the input space, in contrast to more traditional neural network layers. One recent and promising advancement in this field is ML. Still, there is a dearth of studies being carried out in this area. Methods: Therefore, starting with the analysis of magnetic resonance images, we have suggested in this research work a tried-and-tested and methodical strategy for real-time meningioma diagnosis by image segmentation using a very deep transfer learning CNN model or DNN model (VGG-16) with CUDA. Since the VGGNet CNN model has a greater level of accuracy than other deep CNN models like AlexNet, GoogleNet, etc., we have chosen to employ it. The VGG network that we have constructed with very small convolutional filters consists of 13 convolutional layers and 3 fully connected layers. Our VGGNet model takes in an sMRI FLAIR image input. The VGG’s convolutional layers leverage a minimal receptive field, i.e., 3 × 3, the smallest possible size that still captures up/down and left/right. Moreover, there are also 1 × 1 convolution filters acting as a linear transformation of the input. This is followed by a ReLU unit. The convolution stride is fixed at 1 pixel to keep the spatial resolution preserved after convolution. All the hidden layers in our VGG network also use ReLU. A dataset consisting of 264 3D FLAIR sMRI image segments from three different classes (meningioma, tuberculoma, and normal) was employed. The number of epochs in the Sequential Model was set to 10. The Keras layers that we used were Dense, Dropout, Flatten, Batch Normalization, and ReLU. Results: According to the simulation findings, our suggested model successfully classified all of the data in the dataset used, with a 99.0% overall accuracy. The performance metrics of the implemented model and confusion matrix for tumor classification indicate the model’s high accuracy in brain tumor classification. Conclusions: The good outcomes demonstrate the possibility of our suggested method as a useful diagnostic tool, promoting better understanding, a prognostic tool for clinical outcomes, and an efficient brain tumor treatment planning tool. It was demonstrated that several performance metrics we computed using the confusion matrix of the previously used model were very good. Consequently, we think that the approach we have suggested is an important way to identify brain tumors.

## 1. Introduction

Meningiomas, gliomas, and pituitary tumors are common types of brain tumors. Roughly forty percent of intracranial tumors are meningiomas. The 2021 5th edition of the WHO categorization of Central Nervous System cancers (CNS5) builds on the molecular information in meningiomas to aid guide care, while preserving the prior categorization system. Meningioma is categorized into three categories (1–3) by the WHO CNS5 according to molecular profile and histopathological criteria [1]. With three malignancy categories (CNS WHO grades 1–3) depending on histology or subtype, the WHO grading scheme in the WHO CNS5 is therefore comparable to the WHO 2016 edition (Table 1) [2].

The malignancy grading for meningiomas has been altered to a within-tumor grading, regardless of classification, and they are now considered a single tumor category with 15 subtypes. Chordoid and clear cell meningiomas are classified as grade 2 because of their increased propensity to recur. According to the WHO (2016), brain-invasive meningiomas are classified as an atypical meningioma of CNS WHO grade 2 [2] and are generally linked to an elevated risk of recurrence. It is debatable whether those with benign histology and those that have been completely removed behave like grade 2 meningiomas, and the evaluation of brain invasion is subjective and vulnerable to sampling error [3]. While rhabdoid and papillary meningiomas may exhibit more aggressive behavior, these characteristics are no longer enough to classify them as grade 3; instead, they will henceforth be classified as meningiomas in general [4,5].

The subjective evaluation of histological findings is the basis for the malignancy classification of human meningiomas, which makes this approach less than ideal and prone to interobserver variance [6]. Meningiomas of CNS WHO grade 1 with an unanticipatedly early recurrence and meningiomas of CNS WHO grade 2 with a lengthy, slow clinical course without recurrence serve as examples of this [7,8]. The overlap between WHO grade 1 and grade 2 meningiomas in the 2021 classification is primarily seen in terms of clinical behavior and molecular features, rather than strict histological overlap. While most grade 1 meningiomas are slow-growing and benign, some can recur or have a more aggressive clinical course than expected. This is particularly true for those undergoing subtotal resection or having a higher proliferation index (MIB-1). While grade 2 meningiomas are generally atypical with an increased risk of recurrence, some can have a relatively benign clinical course with slow growth and long periods without recurrence [2]. Moreover, some grade 1 meningiomas, especially those with certain genetic alterations like the loss of chromosome 1p or a high MIB-1 index, may behave more like grade 2 tumors. These features are associated with a higher risk of recurrence and may warrant a more aggressive management approach. Some grade 2 meningiomas may lack these features and exhibit a more benign clinical course, making their management a more nuanced decision [9,10]. The overlap highlights the importance of considering both histological grade and molecular features when determining the optimal management approach for meningiomas [11].

In order to improve classification and malignancy grading, the WHO CNS5 book supports molecular biomarkers; however, if conclusive histology of a meningioma subtype is available, molecular biomarkers are not necessary for diagnosis [4]. Numerous genetic abnormalities and driver mutations have been identified by advances in the molecular characterization of meningiomas; Table 2 displays the most notable changes from a clinicopathological perspective.

Meningiomas can therefore be classified as either non-NF2-mutated or NF2 (neurofibromatosis type 2) [12]. Convexity meningiomas are more commonly CNS WHO grades 2 and 3, consist of fibroblastic and transitional phenotypes, and are more frequently NF2-mutated [12]. Non-NF2 meningiomas include meningothelial and secretory phenotypes and are more frequently skull-based [12]. According to genetic analysis, TERTp mutation and homozygous CDKN2A/B loss should be searched for in cases of aggressive atypical meningiomas and meningiomas with borderline grade 2–3 histology. When found, these findings suggest a grade 3 tumor [4,5]. Aggressive behavior is also associated with H3K27me3 loss [13]. It has been demonstrated that DNA methylation may classify meningiomas into methylation groups that more precisely identify patients at high risk of recurrence than histopathology [14]. As a potential future diagnostic work-up for meningiomas, the molecular classification of meningiomas based on copy number variation, point mutations, methylation, and transcriptomic and proteomic data stands out [15].

Although most meningiomas are benign and asymptomatic, the presence of atypical meningiomas and, to a lesser extent, anaplastic ones pose a significant health risk to the patient. The location, size, and growth rate of these tumors can alter brain functions and cause adverse neurological effects depending on the areas of the brain tissue affected, causing symptoms such as headaches, epileptic seizures, visual problems, and coordination problems, among others. Therefore, early detection, precise identification of the grade of malignancy, and timely treatment are vital to improve the patient’s prognosis [16]. A tumor’s prognosis is contingent upon a number of characteristics, including the tumor’s stage of identification, operability, patient age, growth location, and extent of metastasis. Each of these variables affects how the patient responds to radiation and chemotherapy, which in turn affects their chance of survival [1].

The use of magnetic resonance imaging (MRI) is the gold standard for the analysis and diagnosis of meningiomas [17]. The technique known as magnetic resonance imaging (MRI) finds radiofrequency pulses and magnetic flux vectors in the hydrogen atom nuclei of a patient’s water molecules. Since an MRI scan does not use radiation, it is superior to a CT scan from a diagnostic standpoint. However, MRI segmentation and classification of these tumors present challenges that can affect diagnostic accuracy and introduce variability in treatment planning, such as the need for trained personnel, the absence of symptoms, similarity to other intracranial lesions, and subjectivity in the interpretation of neurological images [18]. Inexpensive automated methods are imperative because of the enormous volume of MRI data that needs to be analyzed. Automated cancer identification using MRI is crucial as working with human life requires a high degree of precision. Therefore, improving preoperative classification of meningiomas is a prerogative.

Advancements in artificial intelligence (AI) techniques have significantly improved precision in disease detection and diagnosis. Machine learning (ML), an intersection of statistics and computer science, is a branch of AI as it enables the extraction of meaningful patterns from examples, which is a component of human intelligence. Both supervised and unsupervised ML algorithm approaches can be used to classify brain MR images as normal or diseased. It is possible to classify multi-class publicly available MRI brain tumor datasets quickly without compromising fidelity, allowing for real-time tumor identification [19]. It is also becoming possible to utilize a different strategy that links tumor genetic variation with radiomic characteristics to engender a link between two fields of study, which could be helpful for clinical disease management and improving patient benefits [20].

Our paper offers an efficient automated solution to brain MRI data categorization using ML techniques. Classifying brain MR images is carried out using the supervised ML approach [21,22]. We have employed FLAIR (Fluid-Attenuated Inversion Recovery) structural magnetic resonance imaging (sMRI). Using quantitative and qualitative rendering of different brain subregions, sMRI measures variations in the brain’s water constitution, which are represented as different shades of gray. These data are then utilized to depict and characterize the location and size of tumors. For effective skull stripping (three-dimensional views of brain slices, axial, coronal, and sagittal), FLAIR images guarantee that surrounding fluids are not magnetized and that signals from CSF (cerebrospinal fluid) are suppressed [23,24,25].

The following is a summary of this research work’s major contributions:

- Conventional CT and MRI are reliable for diagnosing meningiomas, with CT demonstrating an accuracy of around 83% [26]. MRI is highly accurate for diagnosing meningiomas, offering sensitivities and positive predictive values generally of 82.6% and above [27]. However, MRI alone may not always differentiate between benign and malignant meningiomas, requiring further investigation like biopsy for a firm diagnosis [28]. Also, while generally effective, MRI accuracy can be lower for smaller lesions or in specific locations like the skull base [27]. Although some researchers have demonstrated that MRI could provide valuable information for the evaluation of meningiomas, the radiological performance of different grades largely overlaps, which could lead to misdiagnosis and inappropriate treatment strategies. Therefore, improving the preoperative classification of meningiomas is a prerogative. ML, an intersection of statistics and computer science, is a branch of artificial intelligence as it enables the extraction of meaningful patterns from examples, which is a component of human intelligence. Over the last decade, it has been successfully applied in the field of radiology, particularly in automatically detecting disease and discriminating tumors. Recently, some studies demonstrated that ML based on MRI was a promising tool in grading meningiomas. However, a few radiomics studies combined with deep learning (DL) features were conducted using a pretrained convolutional neural network (CNN) [29,30].
- This research proposes a novel approach that significantly advances the application of DL for medical diagnostics by employing a very deep transfer learning CNN model (VGG-16) enhanced by CUDA optimization for the accurate and timely real-time identification of meningiomas.
- We have employed FLAIR (Fluid-Attenuated Inversion Recovery) structural magnetic resonance imaging (sMRI) in this case. Using quantitative and qualitative rendering of different brain subregions, sMRI measures variations in the brain’s water constitution, which are represented as different shades of gray. These data are then utilized to depict and characterize the location and size of tumors. For effective skull stripping (three-dimensional views of brain slices, axial, coronal, and sagittal), FLAIR images guarantee that surrounding fluids are not magnetized and that CSF (cerebrospinal fluid) is suppressed.
- In the practice of radiology, error is inevitable. In everyday practice, the amount of evidence collected during the plain film era is thought to be between 3–5% [31]. Interpretative error rates in cross-sectional imaging are reported to be much greater, ranging from 20–30% [32,33]. The clinical implications of accurate brain tumor grading classification are significant, as they can inform treatment decisions and improve patient outcomes. Discussing the method’s integration into clinical workflows has offered insights into its practical applications and impact on patient care, enhancing the paper’s relevance to healthcare professionals, thus providing a practical tool for streamlined medical analysis and decision making.
- A comparison has been made with recent state-of-the-art technique research propositions in the literature review.

## 2. Literature Review

Imaging methods are fundamental to the treatment of cancer and are a great aid in the detection and management of cancers [34,35]. A promising new technique to profile tumors is radiomics, a quantitative approach to analyzing medical pictures that has the potential to provide more individualized care. In order to identify intricate patterns in tumor images that are invisible to the human eye, this method uses mathematical algorithms and artificial intelligence concepts [36,37,38,39]. Radiomic imaging properties may function as biomarkers to monitor and impact clinical endpoints, much like other big data techniques like genomes and proteomics [40]. Radiomics has been shown to have potential in a variety of cancer types. Radiomic models have been applied to the prognostication of distant metastasis and survival, as well as to the identification of cancer subtypes and molecular and genetic variants [41].

Because of the subtle differences in diagnosis, risk assessment, and treatment strategy of meningioma, it is a great oncologic model system for studying the use and development of radiomics in cancer care today [16,42,43]. Traditionally, the World Health Organization (WHO) grading system has guided clinical decision-making for meningiomas [5]. Though some high-grade meningiomas show indolent behavior and may not recur, this grading scheme occasionally reveals significant diversity in tumor behavior, with over 20% of low-grade meningiomas having an early recurrence [44,45,46,47]. This has raised interest in treating patients based on their molecular traits and growth rates [48,49,50,51]. Molecular platforms and testing are not always available, they incur additional costs, and they could only be able to detect a portion of a heterogeneous tumor in practice [52,53]. On the other hand, physicians who treat brain tumors have significantly easier access to imaging. Therefore, if image-based phenotyping can reach high reliability and reflect the biological characteristics and genetic signature of tumors, it has the potential to completely change the way patients can receive precision care.

Brain tumor segmentation is the process of dividing MRI scans into separate segments so that they may be understood more easily. The majority of recent research on CNN application has concentrated on GBM (Glioblastoma Multiforme) segmentation. Using the segmentation process, an image is divided into its individual pixels. This makes it easier to analyze the image and draw conclusions that are insightful. The necrotic core, edema, and enhancing tumor are among the parts of a tumor that this segmentation process correlates to. Since this segmentation entails the process of distinguishing sick tissues from healthy ones, it is essential for precise diagnosis and therapy planning [54]. The initial step in recommending an appropriate course of treatment based on a patient’s response to radiation and chemotherapy is segmentation [55].

For many years, radiotherapists have carried out these operations using manual segmentation. Nonetheless, there is a significant likelihood of variation in the outcomes amongst observers and even within the same observer. The radiologist’s level of skill may have an impact on the laborious process [56]. Segmentation techniques that are automated or semiautomatic have been developed to get around these restrictions. CNNs have been noted as one of the most effective training algorithms, despite processing enormous amounts of data at the expense of substantial computational expenses and complexity [55]. The classification of brain tumors is essential for accurate patient diagnosis and care. However, obtaining appropriate classification is severely hampered by the scarcity of annotated data and the intricacy of tumor images. To increase the performance of brain tumor classification tasks, transfer learning has been a viable method in recent years for utilizing pretrained models on large-scale datasets [57].

In order to improve brain tumor segmentation performance, a research study presented a novel hybrid strategy that blended convolutional neural networks (CNNs) with handmade characteristics. The MRI scans used in this investigation were processed to extract handmade features, such as texture, form, and intensity-based features. Concurrently, a novel CNN architecture was created and trained to automatically identify the features in the data. The handcrafted features and the features found by CNN in various pathways were blended with the suggested hybrid approach to create a new CNN. A range of assessment metrics, including segmentation accuracy, dice score, sensitivity, and specificity, were utilized in this study to gauge performance using the Brain Tumour Segmentation (BraTS) challenge dataset [58]. In a separate investigation, the same research team created a Global CNN (GCNN) that included an additional CNN for MRI Pathways and a CNN for Confidence Surface (CS) Pathways, which handled CS modalities in conjunction with the provided ground truth. The aim of this work was to develop a deep convolutional architecture that is capable of efficiently handling different types of tumors, with the addition of manually created features [59].

In medical imaging, brain tumor segmentation plays a crucial role in detection and therapy while protecting patient privacy and security. Advances in AI-based medical imaging applications are hampered by privacy rules and security issues that often impede data sharing in traditional centralized techniques. Federated learning (FL) was suggested by a research group as a means of addressing these issues. By training the segmentation model on distributed data from several medical institutions without requiring raw data sharing, the suggested approach allowed collaborative learning. Using the U-Net-based model architecture, which is well-known for its remarkable abilities in semantic segmentation tasks, this work highlighted how scalable the suggested method is for widespread use in medical imaging applications [60].

A specific study has additionally taken into account three classification frameworks. The first model applies a fundamental CNN methodology, the second divides it into three categories of brain cancers (glioma, meningioma, and pituitary tumor), and there are two further models: one is normal, and the other exhibits a high degree of metastasis [61]. According to severity, the brain tumor is categorized into three categories (categories I, II, and III) in the third model [61].

Based on information from medical imaging tests, a different study team developed an evolutionary ML algorithm that successfully classifies brain tumor grades. This model, which is a modified version of the recently published Multimodal Lightweight XGBoost [62], is called lightweight ensemble combines (weighted average and lightweight combines multiple XGBoost decision trees).

**Figure 1:**
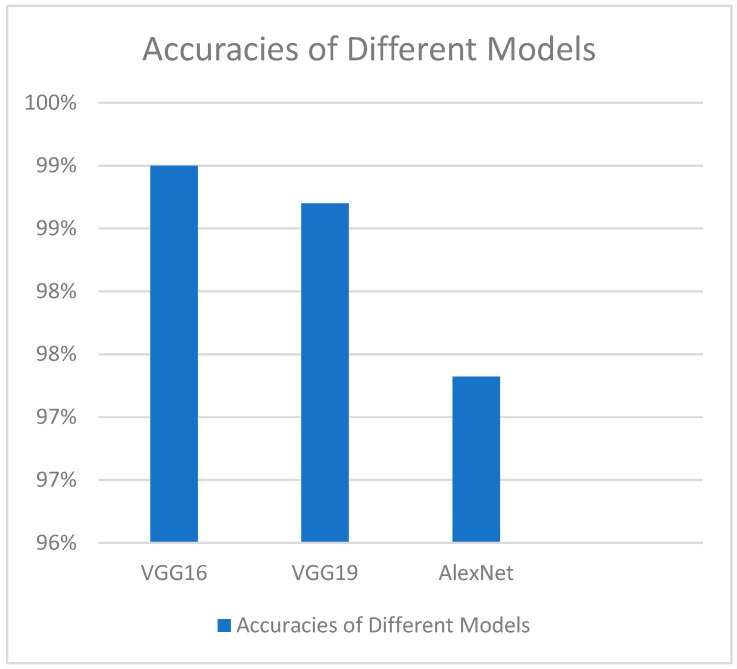
Overall observed accuracies of various deep transfer learning methods.

Different deep transfer learning techniques, such as GoogleNet, InceptionV3, AlexNet, VGG-16, and VGG-19, have been utilized by another study group to determine footprints; InceptionV3 has the best accuracy value but the largest time complexity (Figure 1) [63]. Additionally, it has been noted that, when compared to other models of a similar kind, the GoogleNet model has the steepest learning curve [63].

An additional comparable model has been employed for the prediction of brain tumors, utilizing the transfer learning techniques previously discussed. Additionally, an enhanced freeze strategy has been employed, which modifies the freeze Conv5-AlexNet layer to achieve superior outcomes [64].

Rather than the widely utilized FLAIR images, some proposed study methodologies have used T1-weighted contrast MRI scan images [30]. The application of multi-core GPUs, which generate image pixel matrix multiplication quickly and more efficiently even though it was found to be laborious and time-consuming [65], has represented a significant advancement in the categorization of brain tumors.

Radiomics-based image-based phenotyping has reinterpreted the role of medical imaging. A classic oncologic example that highlights the value of fusing artificial intelligence techniques with quantitative imaging data to enable precision therapy is meningioma.

Globally, meningioma radiomics has surged due to new computational techniques and data accessibility. Research that enhances quality, creates extensive patient datasets, and conducts prospective trials is necessary to ensure translatability into complicated tasks like prognostication [66].

## 3. Methods

### 3.1. Justification for Choosing VGGNet CNN Model

Since it produces a greater level of accuracy than other deep CNN models like AlexNet, GoogleNet, etc., the VGGNet CNN (Convolutional Neural Network) model [67] was chosen. With the aid of three extra 1 × 1 convolutional layers, VGG-16 achieves a 9.4% error rate, which is an improvement over the 9.9% error rate of the prior VGG-13 model [68,69]. Drawing on earlier research, VGGNet has achieved the best results on one of the most difficult datasets ever, the Caltech 256 dataset, which had 30,607 photos in 256 different object categories. This model surpassed numerous others. VGGNet DNN models have been extensively utilized for image classification in a variety of prediction systems for malignancies of the lungs, skin, eyes, breasts, prostate, and other tissues within the past five years [70,71,72,73]. The validity of VGG-16 pertains to the fact that it has been used for transfer learning with pretrained ImageNet weights and to fine tune the MRI images and it has been used widely and proven to be extremely accurate for other detection models like pneumonia, histopathology, and other types of cancers.

### 3.2. Architecture of VGGNet CNN Model

**Figure 2:**
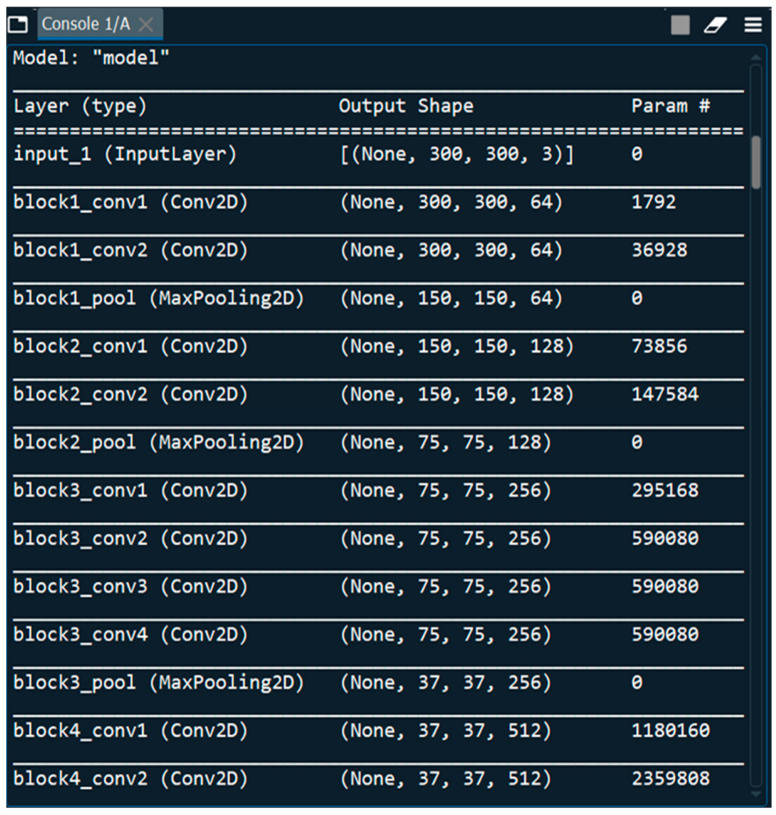

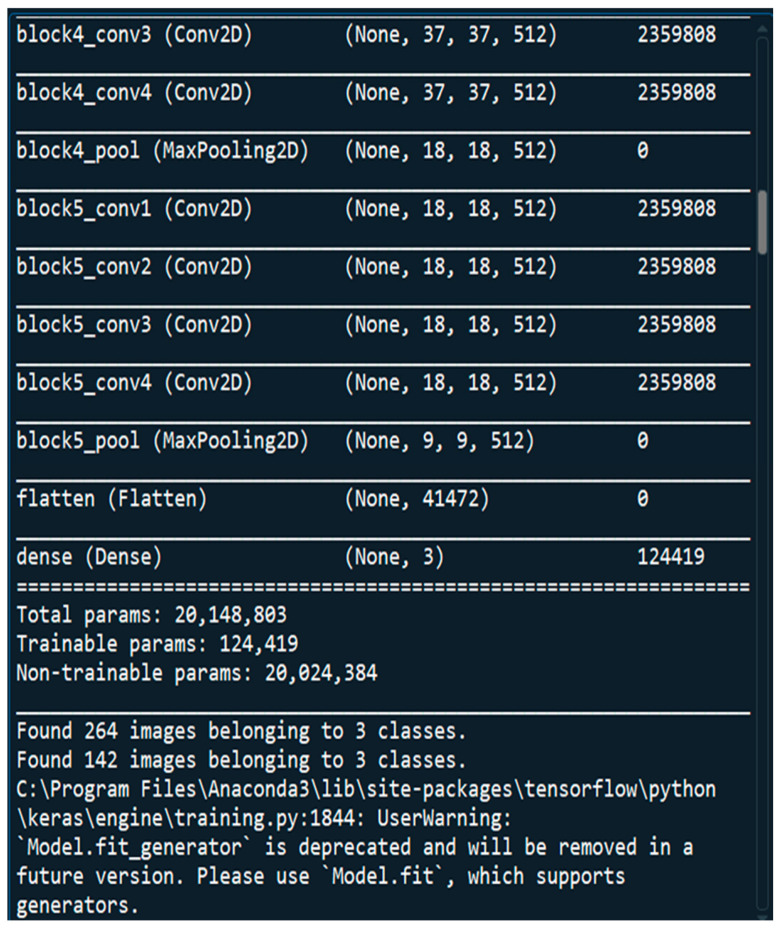
Loading multi-layered VGG-16 DNN Sequential Model.

In our work, we used two Keras models in Python version 3.6, called VGG and Sequential models. VGGNet is a deep learning image pre-processing and classification CNN model that is an improvement on its predecessor, AlexNet (the first famous CNN) [63]. The VGG network that we have constructed with very small convolutional filters consists of 13 convolutional layers and 3 fully connected layers (Figure 2).

Input: Our VGGNet model takes in an sMRI FLAIR image input size of 224 × 224.

Convolutional Layers: VGG’s convolutional layers leverage a minimal receptive field, i.e., 3 × 3, the smallest possible size that still captures up/down and left/right. Moreover, there are also 1 × 1 convolution filters acting as a linear transformation of the input. This is followed by a ReLU unit, which has a piecewise linear function that will output the input if positive; otherwise, the output is zero. The convolution stride is fixed at 1 pixel to keep the spatial resolution preserved after convolution (stride is the number of pixel shifts over the input matrix).

Hidden Layers: All the hidden layers in our VGG network also use ReLU. VGG does not usually leverage Local Response Normalization (LRN) as it increases memory consumption and training time. Moreover, it makes no improvements in overall accuracy.

Fully-Connected Layers: The VGGNet has three fully connected layers. Out of the three layers, the first two have 4096 channels each, and the third has 1000 channels, 1 for each class.

No of Input Images: We have used segments of 264 3D FLAIR sMRI images belonging to three classes (meningioma, tuberculoma, normal). The reason for using a limited number of images is because VGGNet is extremely computationally expensive and takes several days to train on NVIDIA GPU.

A specially developed MRI technique called FLAIR (Fluid-Attenuated Inversion Recovery) MRI highlights regions of tissue with T2 prolongation while suppressing (darkening) the signal from cerebrospinal fluid (CSF). This makes it easier to see brain lesions, particularly in locations near CSF. The goal of FLAIR MRI is to reduce the strong signal from CSF, which can mask mild aberrations in the brain, particularly in regions close to the brain surface and the periventricular region (around the ventricles). It effectively nullifies the CSF signal by using a unique inversion recovery pulse sequence with a long inversion time (TI). In order to generate strong T2 weighting, which identifies regions of tissue T2 prolongation (bright signal), it also uses a long echo duration (TE) [74].

Fluid-Attenuated Inversion Recovery, or contrast-enhanced FLAIR (CE-FLAIR) has demonstrated a high degree of accuracy in identifying meningiomas, particularly those with complete rim enhancement. MRI is a useful tool for meningioma diagnosis, especially for imaging subtle anomalies and outlining tumor margins. With high sensitivity, specificity, and accuracy, the presence of full rim enhancement in CE-FLAIR is a reliable diagnostic of meningioma [75].

**Figure 3:**
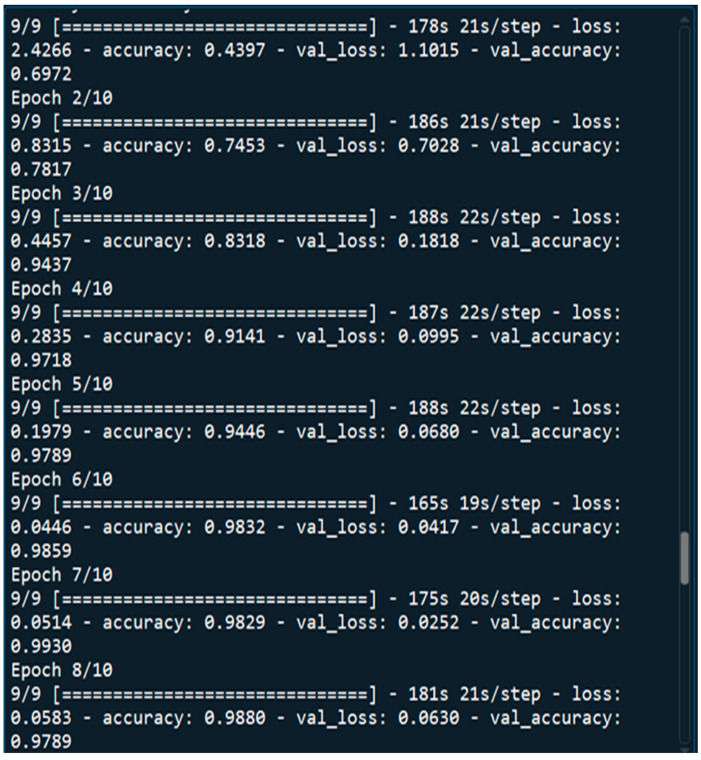

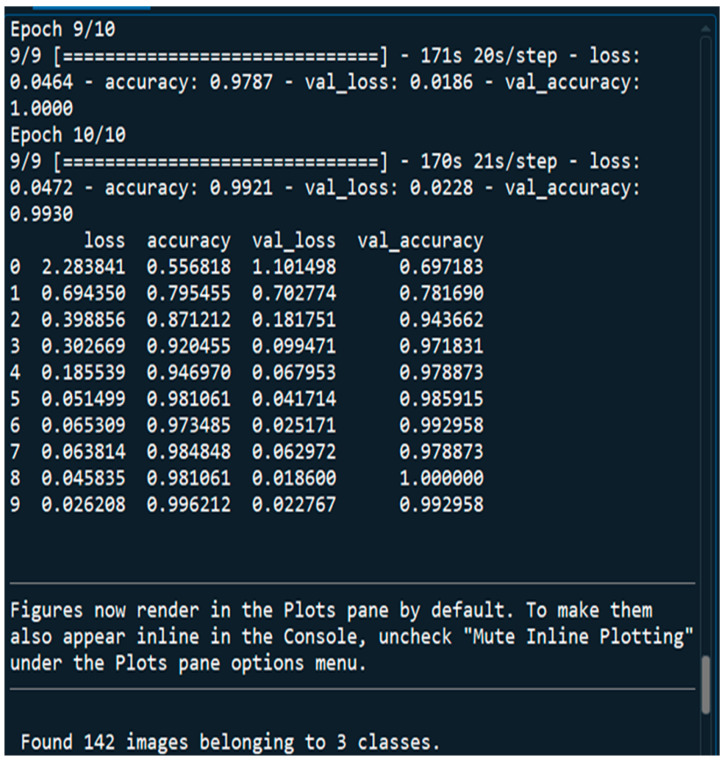
10 Epochs that we considered for training our DNN.

No of Epochs: The number of epochs in the Sequential Model was set to 10 (Figure 3).

The Keras layers that we used are Dense, Dropout, Flatten, Batch Normalization, and ReLU.

In Keras, Dense layers—also referred to as fully-connected layers—are the essential building blocks of neural networks. To add complexity, they first apply linear adjustments to the input data and then apply a non-linear activation function. In deep learning, Dropout is a regularization approach that helps avoid overfitting. During training, it randomly deactivates neurons, pushing the network to pick up more resilient properties. In order to prevent overfitting, the Dropout layer randomly sets input units to 0 at a frequency of rate at each step during the training period. It functions as an ensemble approach, enhances generalization, streamlines training, and improves model performance on unknown data. Input data are reshaped into a one-dimensional array via the Keras “Flatten” layer, enabling neural network interoperability across convolutional and fully connected layers. Enhancing the model’s capacity for generalization and stabilizing the training process are the two main objectives of batch normalization. Additionally, it may lessen the need for meticulous weight initialization of the model and permit the use of faster learning rates, both of which may expedite the training process. In neural networks, Rectified Linear Activation, or ReLU, is a popular activation function. It adds non-linearity, which facilitates the recognition of intricate patterns.

### 3.3. Dataset

The dataset containing MRI scans that we obtained from an anonymous hospital in India contains 264 images belonging to three classes and we considered 14 of them for training and the rest for testing. We used segments of 264 3D FLAIR sMRI images belonging to three classes (meningioma, tuberculoma, normal). The reason for using limited number of images is because VGGNet is extremely computationally expensive and takes several days to train on NVIDIA GPU. We considered implementing the model for more than 5000 images in the future for effective generalization of the problem. All the patients included in this study were aged more than 50 years. The reason for this might be due to the fact that the incidence of meningiomas is highly age-dependent. Meningiomas are most common in the fifth to seventh decades of life (40s to 60s). The average age at diagnosis is around 66 years [76,77].

The first patient is a young, normal healthy patient with no observable pathology. The second patient has meningioma with numerous metastases and the third patient has tuberculoma. The total number of parameters is 20,148,803, with 124,419 trainable parameters and 20,024,384 non-trainable parameters (Figure 2).

### 3.4. Activation Functions

The functions that we used are as follows.

SoftMax—SoftMax is utilized in convolutional neural networks (CNNs) to convert the network’s final layer logits into probability distributions, ensuring that the output values represent normalized class probabilities, making it suitable for multi-class classification tasks.

Considering this function, the function values of both classes are determined and manipulated to add up to unity. This is represented by the undermentioned equation:

$$f_{j}\left(z\right)=\frac{e^{z_{j}}}{\Sigma_{k}e^{z_{k}}}$$

**Cross-entropy loss function**—SoftMax is usually paired with the cross-entropy loss function in the training phase of CNNs. Cross-entropy measures the dissimilarity between the predicted probabilities and the true distribution of the classes. SoftMax, by producing a probability distribution, aligns well with the requirements of the cross-entropy loss function. We exploited this function after utilizing the SoftMax function, whereby our goal was to foresee our network’s performance and, by functioning to decrease this mean squared error, we would be practically optimizing our network. This is represented as follows (both equations are different forms of the same loss function equation):

$$L_{i}=-\log\left(\frac{e^{f_{yi}}}{\Sigma e^{f_{j}}}\right)\;H\left(p,q\right)=-\sum_{x}p(x)\log q\left(x\right)$$

### 3.5. Optimization Algorithm

In this work, we have also compiled the model using the cost optimization technique called the ADAM (Adaptive Moment Estimation) optimizer. ADAM is an adaptive learning rate algorithm designed to improve training speeds in deep neural networks and reach convergence quickly. It customizes each parameter’s learning rate based on its gradient history, and this adjustment helps the neural network learn efficiently as a whole. It uses ⊙, which denotes the Hadamard product (element-wise multiplication), and ⊘, which denotes Hadamard division (element-wise division), while ϵ is the smoothing term used to make sure that division by zero does not take place.

$$m=\beta_{1}m-\left(1-\beta_{1}\right)\nabla_{\theta }J\left(\theta \right)\;s=\beta_{2}s\left(1-\beta_{2}\right)\nabla_{\theta }J\left(\theta \right)⊙\nabla_{\theta }J\left(\theta \right)\;\hat{m}=\frac{m}{1-\beta_{1}^{t}}\;\hat{s}=\frac{s}{1-\beta_{2}^{t}}\;\theta =\theta +\eta {\hat{m}}_{⊘}\sqrt{\hat{s}+\epsilon }$$

### 3.6. DNN Implementation Algorithm

**Figure 4:**
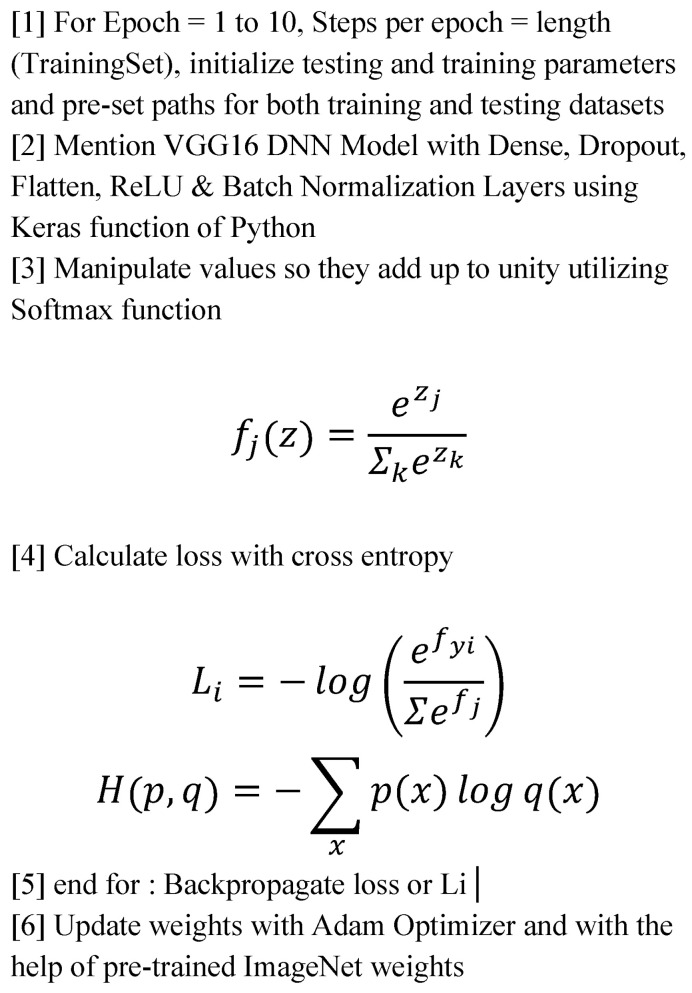
Algorithm for DNN implementation.

We have used a DNN or deep neural network algorithm for this research, which is outlined in Figure 4.

### 3.7. Evaluation of Model Performance

A model’s performance can be judged using a variety of indicators. These measures aid in evaluating how well the model handles false positives and false negatives and predicts the right results. One popular approach for visualizing a model’s performance is a confusion matrix.

**Figure 5:**
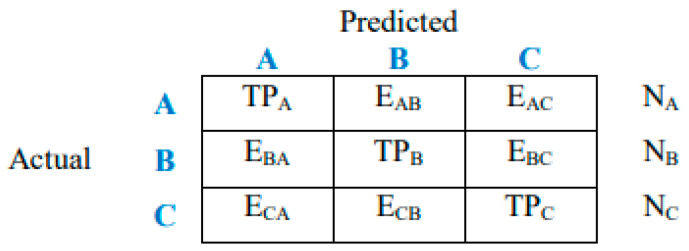
General form of 3 × 3 confusion matrix.

**Figure 6:**
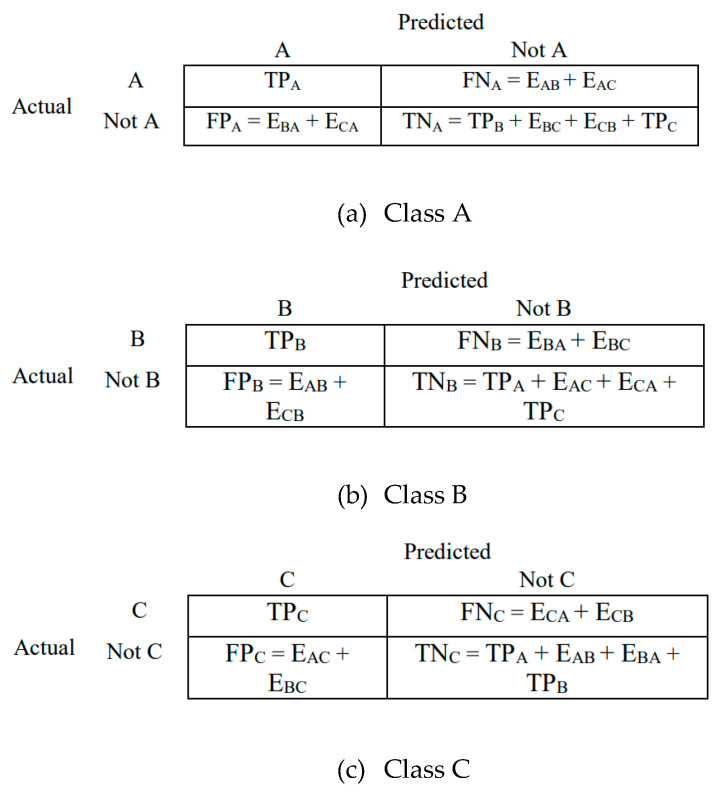
Three 2 × 2 confusion matrices for 3 × 3 confusion matrix shown in Figure 5.

In image processing with a 3 × 3 confusion matrix (Figure 5 and Figure 6), we are evaluating a model that classifies images into three classes. The matrix displays true positives (TPs), true negatives (TNs), false positives (FPs), and false negatives (FNs) for each class, allowing for the calculation of metrics like accuracy, precision, and recall.

Here is a breakdown of understanding the 3 × 3 Matrix.

The rows represent actual classes: each row corresponds to the actual class of an image.

The columns represent predicted classes: each column corresponds to the class predicted by the model.

The cells represent counts: the symbol E_AB_ represents class A samples incorrectly classified as class B, and so on. Each cell shows the number of images that were as follows:

- True positive (TP): Correctly classified as belonging to a specific class.
- True negative (TN): Correctly classified as not belonging to a specific class.
- False positive (FP): Incorrectly classified as belonging to a specific class when it actually belongs to another.
- False negative (FN): Incorrectly classified as not belonging to a specific class when it actually belongs to that class.

In this case, the diagonal elements (TP values) represent the correctly classified instances. The off-diagonal elements represent the misclassifications (FPs and FNs).

Several performance metrics can be derived from the confusion matrix.

Accuracy refers to the ratio of correctly predicted observations to all observations. This is mathematically represented by

$$Accuracy=\frac{TP_{A}+TP_{B}+TP_{C}}{N}$$

Precision refers to the ratio of correctly predicted positive observations to total predicted positive observations. It is related to the false positive rate and is mathematically represented by

$$Precision_{A}=\frac{TP_{A}}{TP_{A}+FP_{A}}$$

$$Precision_{B}=\frac{TP_{B}}{TP_{B}+FP_{B}}$$

$$Precision_{C}=\frac{TP_{C}}{TP_{C}+FP_{C}}$$

Recall refers to the ratio of correctly predicted positive observations to the total observations in the actual class, which is mathematically represented by

$$Recall_{A}=\frac{TP_{A}}{TP_{A}+FN_{A}}$$

$$Recall_{B}=\frac{TP_{B}}{TP_{B}+FN_{B}}$$

$$Recall_{C}=\frac{TP_{C}}{TP_{C}+FN_{C}}\;\;\;\;\;\;$$

The F1 Score refers to the weighted average of precision and recall. It is more useful than accuracy when the class distribution is unequal, or when false positives and false negatives have different costs, and it is mathematically represented by

$$F1_{A}=\frac{2\times Precision_{A}\times Recall_{A}}{Precision_{A}+Recall_{A}}=\frac{TP_{A}}{TP_{A}+0.5(FP_{A}+FN_{A})}$$

Similar expressions are written for classes B and C.

A model’s performance can be assessed using a variety of indicators. A deeper comprehension of the model’s performance and the identification of areas for advancement were obtained in the current work by examining these indicators.

### 3.8. Comparison of the Model with Human Experts

Medical image analysis is complicated, and even if AI algorithms are effective, they might not always be able to pick up on the finer points and subtleties that human experts can. Depending on the data they are trained on, AI systems may exhibit bias. For guaranteeing that the AI’s classifications are precise and trustworthy, a panel of professionals can offer an essential layer of validation, fostering confidence in the technology. Such biases can be recognized and lessened with the use of expert review. A panel of specialists was employed to examine and validate the outcomes of the AI algorithms, making sure that the AI’s interpretations matched human expert knowledge, in order to confirm the correctness of the clinical findings in picture classification. An independent test set of 100 images from 65 patients was used to compare the AI decisions with the decisions made by human experts. A panel of six experts with significant clinical experience in an academic radiology center was instructed to make a decision on each test patient using the patient’s MRI images.

## 4. Output

The fundamental goal of our present study was to develop a well-fitting model, while eliminating underfit and overfit issues. We concluded that our model did not induce overfitting or underfitting. When comparing training and test data, the model loss should be lower in the training data. We discovered that deep learning systems, such as Keras, was advantageous when using neural nets to solve classification tasks.

### 4.1. Performance Metrics Analysis and Discussion

**Figure 7:**
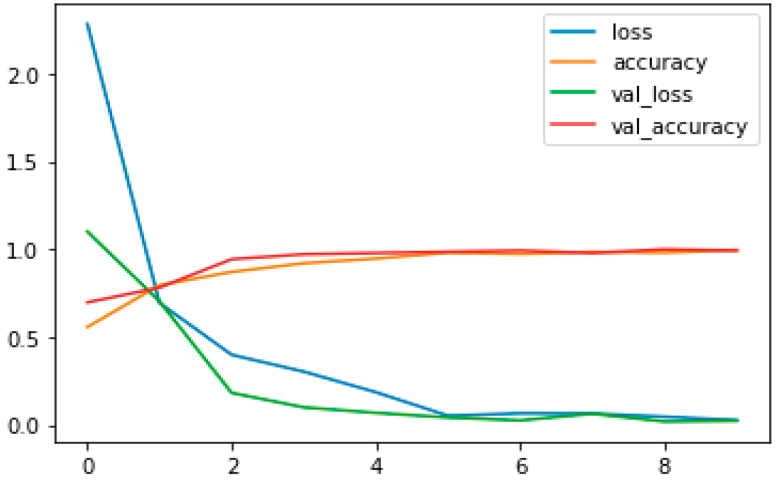
Accuracy and loss curve.

**Figure 8:**
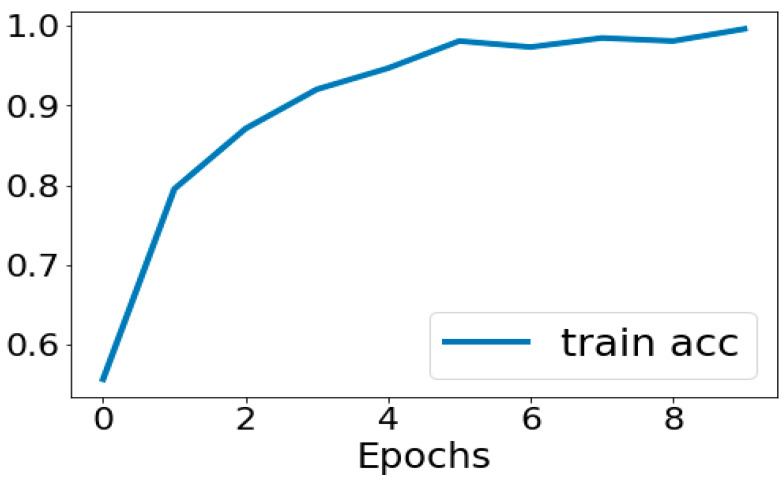
Training accuracy curve.

The performance assessment of the CNN architecture used in the MRI brain tumor image categorization findings is covered in this part. We were able to obtain quite good and reliable results for the predicting capacity for the different types of tumors using the very deep transfer learning CNN that was tested in this work. Our total accuracy is now 99%. The accuracy was obtained using the confusion matrix. There was a panel of six experts. The experts involved had significant clinical experience in an academic radiology center. The analyses were blinded and randomized. Performance was comparable between the AI system and the human experts. The sensitivities and specificities of the experts were plotted on a ROC curve of the trained model, and the differences in diagnostic performance, measured by likelihood ratios, between the model and the human experts were determined to be statistically similar within a 95% confidence interval. The loss and accuracy of our model are displayed in Figure 7, which is plotted below. The training accuracy is seen in Figure 8. These performance charts suggest that the model used did not overfit and that the validation data retained appropriate generalizability with regard to the trained data.

**Figure 9:**
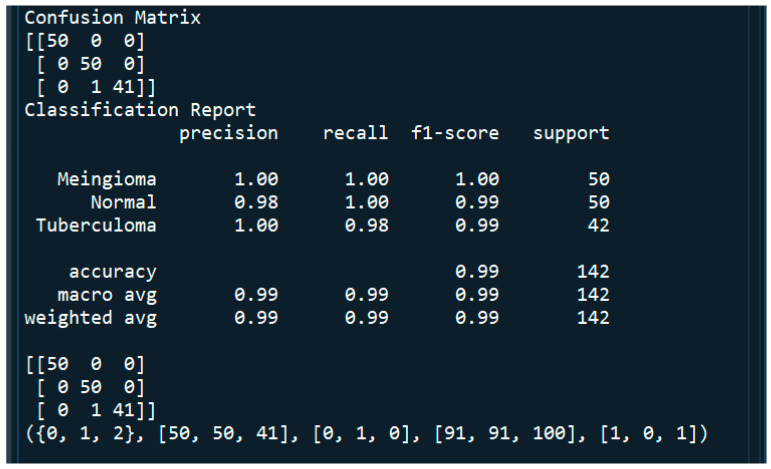
Performance metrics of the implemented model.

**Figure 10:**
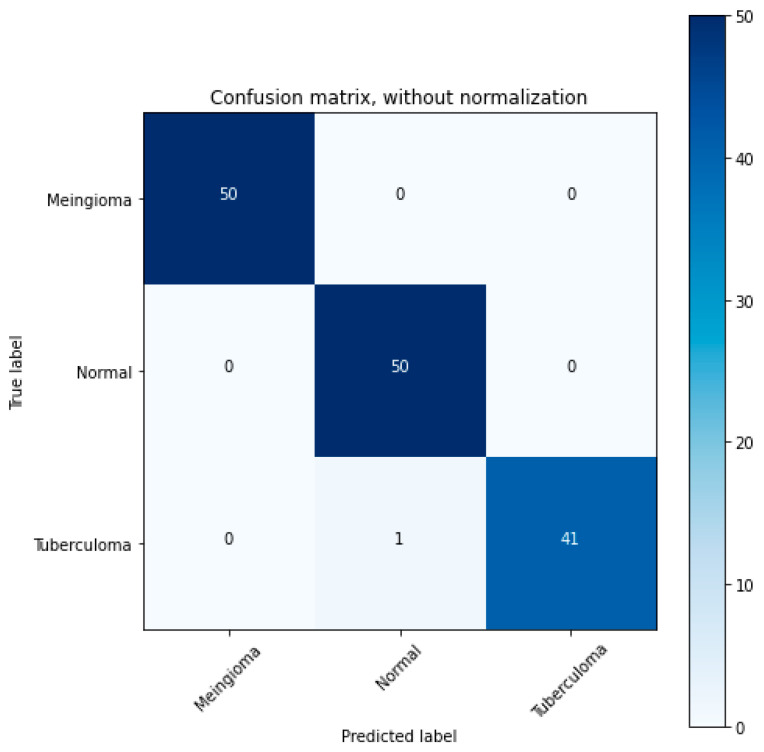
3 × 3 Confusion matrix.

Figure 9 and Figure 10 shows the performance metrics and confusion matrix of the implemented model. The matrix was used to evaluate the VGG-16 model’s performance in brain tumor detection using various metrics to assess its effectiveness. The performance of the VGG-16 model for brain tumor detection was thoroughly evaluated using a comprehensive set of metrics derived from a confusion matrix, including sensitivity, specificity, precision, recall, and the F1 score. The performance of the model is observed to be excellent. The confusion matrix for tumor classification in Figure 9 and Figure 10 indicate the model’s high accuracy in brain tumor classification. The various performance metrics, which are calculated from the confusion matrix of the above implemented model, are as shown in Figure 9 and Figure 10.

The precision score was an important metric used to evaluate the performance of our model. The level of precision obtained can greatly assist clinicians by providing reliable predictions, thus reducing the risk of a false positive diagnosis. The recall score reflects the model’s strong ability to identify all the actual positive cases accurately. A high F1 score signifies a well-balanced model in terms of recall and precision, indicating that our model is neither excessively biased towards false positives nor false negatives. This balance is vital in ensuring a robust and reliable diagnostic model. From the obtained results, it can be inferred that the strong performance metrics demonstrated by our VGG-16 model suggest its significant potential for effective and reliable brain tumor detection from MRI scans. This success opens up avenues for further research into the application of deep learning architectures for medical diagnostic tasks. Future work could involve refining the model and validating its effectiveness on a larger, more diverse dataset, thus paving the way for a reliable, AI-assisted diagnostic tool in neurology.

### 4.2. Comparison of VGG-16 Model Performance with Other Models

The accuracy of the VGG-16 model was compared to a number of other established models in order to provide a comprehensive grasp of its competitive position in the field of brain tumor diagnosis. This comparison with the corresponding accuracies of the models is explained in Table 3.

The performance of the VGG-16 model presents strong competition, even though it does not claim the title of the most accurate model. Our CNN model, which uses a VGG-16 model with CUDA, performs on par with models like GrayNet and the multivariable regression and neural network model, but it has better accuracy than the majority of the other models. The significant potential that the VGG-16 model embodies for the challenge of brain tumor identification is highlighted by this comparative analysis. In order to match or surpass the capabilities of the best models in the field, it is still necessary to pursue more improvements [78,79,80,81,82].

## 5. Conclusions and Future Work

This research proposes a novel approach that significantly advances the application of deep learning for medical diagnostics by employing a very deep transfer learning CNN model (VGG-16) enhanced by CUDA optimization for the accurate and timely real-time identification of meningiomas.

The different test cases that we have observed are summarized in Table 4.

Thus, we were successfully able to test 10 random images and classify them as four “normal” images and six “meningioma” images.

### 5.1. Integration of Output of This Very Deep Transfer CNN Based Real-Time Meningioma Detection Methodology Within the Clinico-Radiomics Workflow

**Figure 11:**
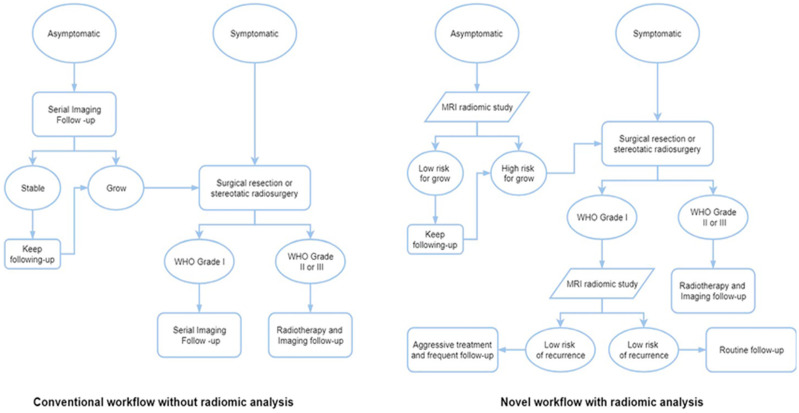
Workflows for various meningioma treatment approaches, both with and without radiomic analysis [83].

Radiomics is a field that extracts and analyzes quantitative features from medical images to improve clinical decision-making. It goes beyond visual assessment by using algorithms to identify subtle patterns and characteristics within images that may be difficult for the human eye to detect. The majority of research utilizing radiomic analysis to investigate meningiomas relied on magnetic resonance imaging (MRI), utilizing one or more imaging sequences. Because different MRI imaging sequences have varying tumor physiology sensitivity, magnetic resonance imaging (MRI) can, in fact, give a superior anatomical delineation (e.g., spatial position) of the cerebral structures and characterize the prevalence of different physiopathological processes [21]. Grade prediction and additional uses are the two main categories into which radiomics applications in meningioma can be broadly classified. For this reason, deep learning-based AI technology such as our implemented model offers previously unheard-of improvements in several medical domains when it comes to automated image analysis. The diagnosis and staging of cancer (preoperative grading), treatment selection, individual treatment optimization, including prognosis modeling, and follow-up imaging are typical areas of use for oncological studies. Numerous imaging modalities can help cancer patients along their path of care [83]. These radiomic results have begun to change the conventional meningioma treatment workflow (Figure 11).

Although there are four basic processes in the radiomics workflow (image acquisition, segmentation, feature extraction, and statistical analysis/model) [38,84,85], each step varies slightly depending on the study and its goals [39].

The process of radiomics workflow commences with image acquisition, which involves obtaining and reconstructing the image data [39]. The region of interest (ROI) is identified and segmented in the second stage, which can be conducted manually, automatically, or semiautomatically. Meningiomas are typically manually defined by skilled radiologists in clinical situations. Because many other tumor types lack well-defined borders and internal heterogeneity, the resulting inter-user variability is unavoidable. While there are other approaches to reducing variability, segmentation techniques are a common one [85].

Choosing segmentation software wisely and manually verifying results with vision can improve the outcome and increase workflow efficiency, particularly when a radiologist is managing hundreds of cases concurrently. Generally speaking, the human brain is not as efficient as a computer when it is tired, anxious, or has limited expertise. This might lead to misdiagnosis or missing a lesion during an MRI. In contrast, artificial intelligence (AI) may deliver dependable results in a short amount of time, making up for human limitations and avoiding mistakes in clinical settings. Therefore, in this radiomic workflow stage, our AI-enabled system can be helpful for novices learning MRI as well as for professionals who are tired or for negligence brought on by people who have had a lot of screenings. In some circumstances, alternative strategies like segmenting a fixed-size ROI [86] or applying an algorithm [87] might also be effective.

Decoding and quantitatively outputting the high-dimension image data is the next stage of feature extraction [66]. Currently, feature extraction patterns may be easily categorized as either having human commands or not [63]. The traditional method requires specialized algorithms that are run by humans. However, the more recent mode, which is based on deep learning radiomics (DLR) and uses CNNs as an example, like our model in this workflow stage, can almost entirely carry out the remaining tasks automatically and without the assistance of humans. Furthermore, compared to conventional approaches, the number of recovered features from CNNs is many orders of magnitude higher [63]. However, in order to prevent overfitting, feature dimensions must be reduced [66]. Additionally, several layers inside a single CNN can be used for feature extraction, selection, and classification [63]. Semantic and agnostic features make up the two categories of radiomics features. The radiology lexicons that are frequently employed to intuitively define the lesion, such as size, location, and shape, are indicated by semantic characteristics. In contrast, agnostic features are quantitative descriptors that are derived theoretically with the intention of emphasizing lesion heterogeneity [66]. There are three types of agnostic features: first-, second-, and higher-order features. First-order statistics, which are usually based on the histogram and show skewness and kurtosis, show the distribution of values of individual voxels without taking into account spatial correlations. Second-order statistics characterize statistical correlations, or “texture” features, between voxels that have comparable (or dissimilar) contrast values. Higher-order statistical features, such as Laplacian transforms, Minkowski functionals, etc., are repeating or nonrepetitive patterns filtered through particular grids on the picture [87].

The chosen features can be utilized for a variety of analyses in the last stage of statistical analysis and modeling, and they are typically included into predictive models to offer better risk stratification [39]. The process of creating a model involves integrating a number of analysis techniques, grouping features, and allocating distinct values to each feature based on the information content that has been predetermined. These analytical techniques will make use of statistical techniques, ML, and artificial intelligence. A perfect model will be able to handle sparse data, such as genetic profiles, in addition to handling the extracted features well [87]. The model’s versatility increases with the number of covariates it can manage.

In order to accurately classify meningiomas from MR image slices, a deep learning architecture utilizing CNNs was discussed and put into practice in this study. In the end, better patient outcomes may result from this research’s ability to provide more accurate and customized treatment strategies for patients with correctly diagnosed brain tumors.

### 5.2. Limitations of Our Study

Still, there is always space for improvement, and further research is needed in a number of areas. One of the study’s methodological flaws is data bias, which is the predominant representation of data from a single age group (those > 50) and may result in a lack of diversity in the dataset and restricted generalizability to other datasets and imaging modalities. The reason for this might be due to the fact that the incidence of meningiomas is highly age-dependent. Meningiomas are most common in the fifth to seventh decades of life (40s to 60s). The average age at diagnosis is around 66 years [76,77]. The whole population should have been included in a dataset that was well-balanced.

We used segments of 264 3D FLAIR sMRI images belonging to three classes (meningioma, tuberculoma, normal). The reason for using a limited number of images is that VGGNet is extremely computationally expensive and takes several days to train on NVIDIA GPU. A limited number of images can negatively impact the generalization potential of a VGG-16 CNN model, especially when training from scratch, leading to underfitting and poor performance on unseen data. However, transfer learning with a pretrained VGG-16 model can mitigate this issue, allowing for faster training and improved generalization. We have considered implementing the model for more than 5000 images in the future for effective generalization of the problem.

Its incapacity to differentiate between various brain tumor subtypes is another drawback. Meningiomas are smaller than gliomas, and the former are frequently more noticeable than the latter. Gliomas, however, can readily pass for meningiomas in MRI because of characteristics including the broad dural contact, CFS cleft sign, and dural tail sign, which could lead to confusion in the diagnosis. High-grade glioma invading the dura mater may also be difficult to distinguish from meningioma, as both lesions show high relative cerebral blood volume (rCBV) values in perfusion MRI. In these cases, the evaluation of the time–intensity curve was shown to be a helpful approach. In MR spectroscopy, an elevated distinct metabolite peak at 3.8 ppm may allow a differentiation between meningiomas, high-grade gliomas, and intracranial metastases [17].

Correct brain tumor grading classification has important therapeutic ramifications since it can guide therapy choices and enhance patient outcomes. The ultimate goal of this project is to accurately detect brain tumors at the medical picture analysis stage and throughout the planning and execution of robotic surgery by using deep learning techniques in computer vision.

#### Computational Complexity of the Model: Trade-Offs Between Accuracy and Computational Demands

CNNs’ deep architectures are typically arranged in a pipeline of descriptors that progresses in representational granularity from edges and corners to motifs, sections, and, at the end, objects. In order to accomplish this, CNNs rely on fully connected and Softmax classification modules to deliver the final classification of the input image, as well as many convolutional and sub-sampling layers that acquire the features and reduce their dimensionality, respectively. For these reasons, a high computational burden and memory occupation are common characteristics of CNNs. With more than 138 million parameters that demand more than 527 MB of storage and more than 13 billion operations for processing the input image, VGG-16 is computationally and memory intensive [88].

Hence, the computational cost of the VGGNet model is very high. On an NVIDIA GPU, training takes many days. Therefore, we were motivated to add more input photographs to the model after implementing it for 264 images in the beginning. The testing and training data were split into 60% and 40% sets. We used a total of 20,148,803 parameters, of which 124,419 were trainable and 20,024,384 were not, in order to balance the trade-offs between accuracy and computing needs. Increasing the Imagenet accuracy for a range of parameter values is something we are excited about.

### 5.3. Potential Future Research Directions

We intend to continue developing the Inception-V3 model in the future. Moreover, additional research could be conducted to categorize these images as normal, gliomas, pituitary tumors, tuberculomas, and meningiomas, and this process would also be able to determine the extent of metastasis rather than relying solely on a basic prediction system that distinguishes between normal results and meningiomas. The grade of the tumor can also be used to classify it. Current developments in this field of study include the application of more effective methods that need less time complexity and have higher accuracy. Several layers of CNNs are altered to improve performance. Future research should focus on exact tumor delineation and characterization, generative AI, massive medical language models, multimodal data integration, and racial and gender inequities [28,89]. Clinical results will be optimized by personalized treatment techniques that are adaptive. Like RNNs (Recurrent Neural Networks), integrated techniques can be used in conjunction with a SVM (Support Vector Machine) to improve detection. Subsequent research endeavors ought to concentrate on integrating the auspicious outcomes of ML algorithms into clinical settings, encompassing the creation of software solutions that are easy to employ for medical practitioners. In the future, robotic neurosurgery could greatly benefit from the integration of AI with cutting-edge technologies like neuronavigation and augmented reality, ushering in a new era of surgical procedures that are safer and more precise.

Terminologies: meningioma, glioma, pituitary, metastasis, cross entropy, deep learning, convolutional neural networks, transfer learning, radiomics.

## Figures and Tables

**Table 1:** Meningioma subtypes.

Histological Type	Histological Malignancy Grade
Meningothelial meningioma	1/2
Fibrous meningioma	1/2
Transitional meningioma	1/2
Psammomatous meningioma	1/2
Angiomatous meningioma	1/2
Microcystic meningioma	1/2
Secretory meningioma	1/2
Lymphoplasmacyte-rich meningioma	1/2
Atypical meningioma (including brain infiltrative meningiomas)	2
Chordoid meningioma	2
Clear cell meningioma	2
Anaplastic (malignant) meningioma	3

**Table 2:** Clinicopathologically significant genetic changes in meningiomas in humans.

Genetic Alteration	Clinicopathological Significance
*NF2* mutation	Convexity meningiomas, fibrous, and transitional subtypes, more often CNS WHO grade 2/3
*TRAF7* mutations	Secretory subtype
*TERT* promotor mutation	CNS WHO grade 3
*SMARCE1* mutation	Clear cell subtype
*BAP1* mutation	Rhabdoid and papillary subtypes
*CDKNA2A/B* loss	CNS WHO grade 3
*H3K27me3* loss	Increased risk of recurrence
DNA methylation profiling	Methylation classes associated with increased risk of recurrence

**Table 3:** Comparison of model accuracies.

Model	Accuracy
VGG-16 (our model)	99%
EasyDL	96.6%
GoogLeNet	92.54%
GrayNet	95%
ImageNet	91%
CNN	96%
Multivariable Regression and Neural Network	95%

**Table 4:** Test cases with their prediction results.

Test Case No.	Prediction Result
1	Meningioma
2	Meningioma
3	Meningioma
4	Meningioma
5	Meningioma
6	Meningioma
7	Normal
8	Normal
9	Normal
10	Normal

## Data Availability

The data presented in this study are available upon reasonable request from the corresponding author. The proposed code and dataset will be uploaded soon in either GitHub or Kaggle (latest versions).

## Abbreviations

WHO CNS5	The 2021 World Health Organization Classification of Tumors of the Central Nervous System, is an update to the previous 2016 classification. It incorporates findings from the Consortium to Inform Molecular and Practical Approaches to CNS Tumor Taxonomy (cIMPACT-NOW) and emphasizes the role of genetic and molecular changes in tumor characteristics. This fifth edition includes new tumor types, revised nomenclature, and refined grading systems
sMRI	structural Magnetic Resonance Imaging is a non-invasive imaging technique for examining the anatomy and morphological pathology of the brain,
CUDA	Compute Unified Device Architecture is a parallel computing platform and application programming interface (API) developed by NVIDIA for general computing on Graphical Processing Units (GPUs) with dramatic escalation of computing application speeds,
CNN	Convolutional Neural Network is an advanced version of artificial neural networks (ANNs), primarily designed to extract features from grid-like matrix datasets. This is particularly useful for visual datasets such as images or videos, where data patterns play a crucial role,
FLAIR	Fluid-Attenuated Inversion Recovery MRI highlights regions of tissue with T2 prolongation while suppressing (darkening) the signal from cerebrospinal fluid (CSF). This makes it easier to see brain lesions, particularly in locations near CSF. The goal of FLAIR MRI is to reduce the strong signal from CSF, which can mask mild aberrations in the brain, particularly in regions close to the brain surface and the periventricular region (around the ventricles). It effectively nullifies the CSF signal by using a unique inversion recovery pulse sequence with a long inversion time (TI). In order to generate strong T2 weighting, which identifies regions of tissue T2 prolongation (bright signal), it also uses a long echo duration (TE),
CSF	Cerebrospinal Fluid which is a clear, colorless fluid that surrounds the brain and spinal cord, acting as a cushion and providing nutrients and waste removal,
GBM	Glioblastoma multiforme, also known as glioblastoma, is the most common and aggressive type of primary brain tumor in adults,
ReLU	Rectified Linear Unit is one of the most popular activation functions for artificial neural networks, and finds application in biomedical image processing, computer vision and speech recognition using deep neural nets and computational neuroscience,
DNN	Deep neural networks are a type of artificial neural network with multiple hidden layers, which makes them more complex and resource-intensive compared to conventional neural networks. They are used for various applications and work best with GPU-based architectures for faster training times,
ADAM	Adaptive Moment Estimation optimizer is an adaptive learning rate algorithm designed to improve training speeds in deep neural networks and reach convergence quickly. It customizes each parameter’s learning rate based on its gradient history, and this adjustment helps the neural network learn efficiently as a whole,
RNN	Recurrent Neural Network is a type of deep learning model specifically designed to process sequential data like text, speech, or time series,
SVM	A Support Vector Machine is a supervised machine learning algorithm used for both classification and regression tasks. It works by finding the optimal hyperplane that separates data points into different classes, maximizing the margin between them. SVMs are particularly effective for binary classification and can handle both linear and non-linear data using kernel functions,
VGG-16	refering to Visual Geometry Group of the University of Oxford, is a convolutional neural network (CNN) model primarily used for image classification and object recognition. It’s known for its simplicity and effectiveness, making it a foundational model in the field of computer vision. The “16” in VGG16 refers to the number of layers in the network that have learnable parameters, including convolutional and fully connected layers,
DLR	Deep Learning Radiomics is a fusion of deep learning and radiomics which are powerful techniques for extracting and analyzing quantitative features from medical images, enabling precision imaging in various applications. These techniques can help in diagnosis, prognosis, and treatment planning by identifying patterns and biomarkers that might not be readily apparent to the human eye.
